# Seasonal Prevalence of the Invasive Longhorn Beetle *Aromia bungii* in Osaka Prefecture, Japan

**DOI:** 10.3390/insects13030222

**Published:** 2022-02-23

**Authors:** Yuichi Yamamoto, Shuji Kaneko

**Affiliations:** Research Institute of Environment, Agriculture and Fisheries, Osaka Prefecture, 442 Shakudo, Habikino 583-0862, Japan; kanekos@mbox.kannousuiken-osaka.or.jp

**Keywords:** *Aromia bungii*, body size, Cerambycidae, emergence, invasive species, longhorn beetle, Rosaceae, seasonal prevalence, sex ratio, wood borer

## Abstract

**Simple Summary:**

Recently, damage caused by the invasive longhorn beetle *Aromia bungii* in Rosaceae trees, such as ornamental cherry, peach, and Japanese apricot, has become a serious problem in Japan, indicating the need to establish an effective control program against the beetle. To determine the optimal timing for pest control, we surveyed the seasonal abundance of the adult beetle based on the number of adults sighted on host trees over 3 years at three study sites (one site from 2019–2021 and two sites from 2020–2021) in Osaka Prefecture, Japan. The field surveys revealed that adult appearance periods spanned 2 months (from June to August) and that the peak sightings occurred in late June. The adults were more abundant in the field in the early phase of the appearance periods. These results will help in the timely control of *A. bungii* adults to reduce their population density in the field. An optimal timing of application is near the peak day, which occurs in late June in Osaka Prefecture. Because seasonal adult abundance varies among regions, it is important to investigate each invaded region.

**Abstract:**

A thorough understanding of the seasonal prevalence of invasive pests in newly invaded regions is key for establishing an appropriate and localized control plan for their successful eradication. In this study, we investigated the seasonal prevalence of the invasive longhorn beetle *Aromia bungii* (Coleoptera: Cerambycidae) in Osaka Prefecture, Japan. We determined the number of adult beetles sighted on host trees more than once a week from late May or early June to late August for 3 years at three study sites (one site from 2019–2021 and two sites from 2020–2021). The appearance period of *A. bungii* adults spanned over 2 months (June–August), and peak sighting in the field occurred in late June; the adults were more abundant in the early phase of their seasonal prevalence (around the peak dates) and almost disappeared by August. The number of adult beetles emerging from *A. bungii*-infested trees at one study site was surveyed daily in 2021. This survey showed a short-span adult emergence period: Approximately 1 month from the first emergence day, supporting the idea of the concentration of adult abundance in the early phase. These results will help to establish a timely pest-control plan for *A. bungii* in Osaka Prefecture.

## 1. Introduction

The red-necked longhorn beetle *Aromia bungii* (Faldermann, 1835; Coleoptera: Cerambycidae) is an invasive pest that infests the Rosaceae family [1,2,3]. In recent years, the invasion and establishment of *A. bungii* have been reported in various regions across Japan [4,5]. The beetle attacks and damages the host tree including ornamental cherry, peach, Japanese apricot, and Japanese plum, severely impacting urban greenings and fruit-tree industries in the affected regions [5,6]. Accordingly, tree owners have implemented various measures in response to *A. bungii*, such as the removing infested trees, injecting insecticides against larvae in the trunks, and spraying insecticides against adults in the field [6,7].

The seasonal prevalence of target pests acts as useful information for planning pest-control strategies. Notably, dates with a higher adult abundance (the peak dates) and adult appearance periods in the field are ideal times and durations for the application of control measures, respectively. In Japan, *A. bungii* generally has a 2-year life cycle [4], and adults can be sighted from June to August in the field [7]. Previous field surveys in Japan have suggested that the peak dates and appearance periods of *A. bungii* adults vary by region, but few detailed surveys have been conducted, which took place in early July and mid-June to early August in Itano-Cho, Tokushima Prefecture [8], in early July and late June to mid-July in Soka City, Saitama Prefecture [9], and in mid-July and late June to mid-August in Tatebayashi City, Gunma Prefecture [4]. The appearance periods of *A. bungii* adults also vary across regions [4] in its native country China [1]. This regional difference has also been reported in other cerambycids in Japan [10,11]. Moreover, seasonal prevalence in a region has been reported to vary annually [12,13]. Considering this expected variation, a multi-year and multi-site survey in a region can help to identify a general trend of seasonal prevalence in the region; however, such surveys have rarely been conducted for *A. bungii* adults.

Field surveys on the seasonal prevalence of longhorn beetles have mostly been conducted using pheromone lure traps [14,15,16] or personal direct sampling [11,13,17,18]. However, the practical use of trap apparatus with pheromone lure blends has not yet been realized for *A. bungii* [19,20,21]. As such, previous field surveys for seasonal prevalence in Japan [4,8,9] were conducted by direct sampling.

Similar to surveys conducted to determine seasonal prevalence, surveys for emergence forecasting, such as initial emergence dates, have been conducted through daily record-keeping of adult cerambycid emergences using infested logs carried inside outdoor net chambers [11,17,22,23,24]; however, in *A. bungii*, infested logs have been used for not emergence forecasting but biological studies thus far [3,25,26]. Emergence timings via infested logs surveys may be influenced by the environmental conditions of the places where the net chambers are located [10] and may not accurately reflect those in the field. Therefore, the direct use of infested trees in the field [27] may reflect field conditions more accurately.

The matured larvae of *A. bungii* create holes in infested trees 1 year before their emergence, a phenomenon called “planned adult emergence hole” [4,7]. Accordingly, we could record the emergence dates of *A. bungii* adults using infested trees located in the study area by setting up nets on the holes to capture the emerging adults. Data on the individual emergence date of *A. bungii* adults from infested trees may provide evidence for the seasonal prevalence of *A. bungii* in the field. In addition, factors that affect their emergence dates, such as sex and body size, can be examined using the data.

This study aimed to identify the seasonal prevalence of *A. bungii* in the field in Osaka Prefecture. We surveyed the number of *A. bungii* adults sighted on host trees during late May or early June to late August from 2019 to 2021 at three study sites―one study site from 2019 to 2021 and two study sites from 2020 to 2021. Additionally, we surveyed the emergence dates of *A. bungii* adults from infested trees in the field on a daily basis and recorded their characteristics (sex and body size) at one study site in 2021. Based on the characteristics data, we analyzed factors affecting the emergence timings of individual adults using a linear mixed model (LMM). These data of the seasonal prevalence of *A. bungii* can provide valuable information for planning a localized pest-control strategy in Osaka Prefecture.

## 2. Materials and Methods

### 2.1. Survey Years and Study Sites

**Figure 1:**
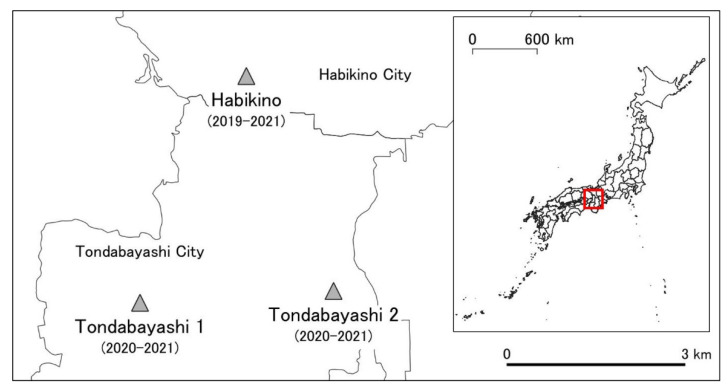
Three study sites (Habikino, Tondabayashi 1, and Tondabayashi 2) to determine the seasonal prevalence of *Aromia bungii* in the field in Osaka Prefecture, Japan. Shaded triangles denote the locations of the study sites. The numbers in parentheses represent the survey years. The map in the top right corner is a map of Japan’s prefectures. The red square represents the location of the study sites (Osaka Prefecture).

The study sites were located approximately 3 km apart from each other and were selected by considering the estimated annual migration distance of *A. bungii*—approximately 2 km on average and a maximum of 3 km [28]—so that adult populations among the study sites did not mutually interact during the current year’s surveys. The survey years and study sites in Osaka Prefecture were as follows:

1. Research Institute of Environment, Agriculture and Fisheries, Habikino City, Osaka Prefecture (34°32′8″ N, 135°35′53″ E) (hereafter referred to as Habikino) from 2019 to 2021.
2. Sidewalk along Route 309 in Tondabayashi City (34°30′22″ N, 135°34′33″ E) (hereafter referred to as Tondabayashi 1) from 2020 to 2021.
3. Riverside road of Ishikawa River in Tondabayashi City (34°30′9″ N, 135°36′50″ E) (hereafter referred to as Tondabayashi 2) from 2020 to 2021 (Figure 1).

### 2.2. Determination of the Number of Adults Sighted on Trees in the Field

We predetermined the survey routes at each study site prior to the start of each year’s survey. The survey routes included a minimum of 100 monitoring trees to ensure adequate opportunities for encounters with *A. bungii* adults. Regarding the monitoring trees, we selected all the host trees planted in series along the predetermined routes, except those that were buried in shrub and were inaccessible. At each study site, dead or weakened trees were cut annually after the current year’s survey was completed. Therefore, we reselected the survey routes (i.e., monitoring trees) annually prior to the start of the next year’s survey. In some cases, the monitoring trees were cut or were no longer accessible before completing the current year’s surveys. These survey routes (i.e., monitoring trees) were not changed in the middle of the current year’s surveys. The number of monitored trees in each survey year and study site is shown in Appendix A (Table A1).

We commenced the field surveys in late May or early June and completed them by late August, in accordance with the previous research season in Japan [8]. We mainly surveyed in clear weather conditions, but occasionally on cloudy or rainy days. Because the June–July period corresponded to the rainy season in the region, we occasionally encountered unfavorable weather conditions during the surveys. The frequency and time zone of our survey were more than once a week between 11:00 a.m. and 16:00 p.m., respectively.

We walked along the survey routes and searched for *A. bungii* adults on the monitoring trees, mainly focusing on tree trunks and main branches from the ground to a visible height (approximately 3 m). We recorded both the number and sex of adult beetles sighted on the monitoring trees. Most of the sighted adults were captured by hand or using sweeping a net and transported to the laboratory of the Research Institute of Environment, Agriculture and Fisheries, Osaka Prefecture, using registered containers for invasive alien species (permission No. 18000285, Kinki Regional Environmental Office) for use in insecticidal efficacy tests against *A. bungii* adults; however, we will not report the results here.

Seasonal prevalence was assessed based on the total number and number of males and females of sighted adults per survey day in each survey year and study site. Among the non-captured adults, some were overlooked in terms of sex and were thus not included in the visualization for sex.

### 2.3. Determination of Emergence Dates of Emerged Adults from Infested Trees in the Field

**Figure 2:**
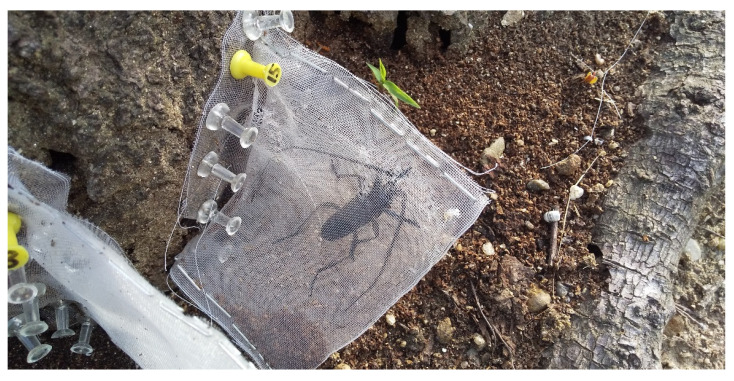
An emerged *Aromia bungii* adult captured in a fine-mesh net set over an emergence hole using push pins and staples on a tree stump on 14 June 2021.

To determine the emergence dates of *A. bungii* adults from infested trees, we selected nine infested trees (four standing trees and five tree stumps) (*Cerasus × yedoensis* ‘Somei-yoshino’) at Habikino study site in December 2020. The selected trees had planned adult emergence holes with an oval shape typical for *A. bungii* [4,7] in the winter, which indicated that the *A. bungii* adults would emerge through the holes during the next year’s emergence period. We set fine-mesh nets (Sunsunnet SL 4200, Nihon Widecloth Co., Ltd., Osaka, Japan) using push pins and staples over a total of 80 holes in the nine test trees (Figure 2) on 22 May 2021. We conducted daily surveys, with or without adult capture, between 23 May and 31 August (twice per day, at approximately 9:00 a.m. and 17:00 p.m. from 1 June to 31 July) and recorded the date and sex of the captured adults. We then brought the adults to the laboratory on the same day. The forewing lengths of the beetles were measured using an electronic caliper (Absolute Digimatic Caliper CD-20APX, Mitutoyo, Kanagawa, Japan). The net-capture date was considered the emergence date of the adults. The number of adults captured per day was visualized for each tree.

### 2.4. Statistical Analyses

All statistical analyses and data visualizations were performed using R software version 4.0.3 [29]. The packages “lme4,” “multcomp,” and “MuMIn” were used for the analysis, and “ggplot2” and “cowplot” were used to plot and visualize the data.

To determine the variable factors affecting the emergence day of net-captured *A. bungii* adults, we applied LMM (“lmer” function of the “lme4” package) to explain the number of elapsed days since the date of first emergence of *A. bungii* (response variable) among the captured adults. The probability distribution of the response variable was assumed to be a Gaussian distribution. The explanatory variables in the full model were as follows: forewing length (mm) as an indicator of body size (continuous variable), sex (two categories: male or female), and tree type (two categories: standing tree or tree stump). We performed model selection for the explanatory variables on a full model under the random intercept on each test tree as a random effect term using the maximum likelihood method. The most predictive model was selected among the models with all combinations of the explanatory variables (“dredge” function of “MuMIn” package) using the smallest Akaike’s information criterion (AIC) value [30]. To check the validity of using the LMM but not the linear model (LM), we calculated the deviance of both models with the same explanatory variables based on the most predictive LMM.

The results of the model selection for explanatory variables with coefficients of the selected variables were compared to those of the models with the second smallest AIC value. Multiple comparisons of the difference of the estimated marginal means between the categories within each fixed effect were performed using the Tukey–Kramer honestly significant difference (HSD) test (*p* < 0.05) (“glht” function of “multcomp” package).

## 3. Results

### 3.1. Seasonal Prevalence Based on the Number of Sighted Adults in the Field

**Figure 3:**
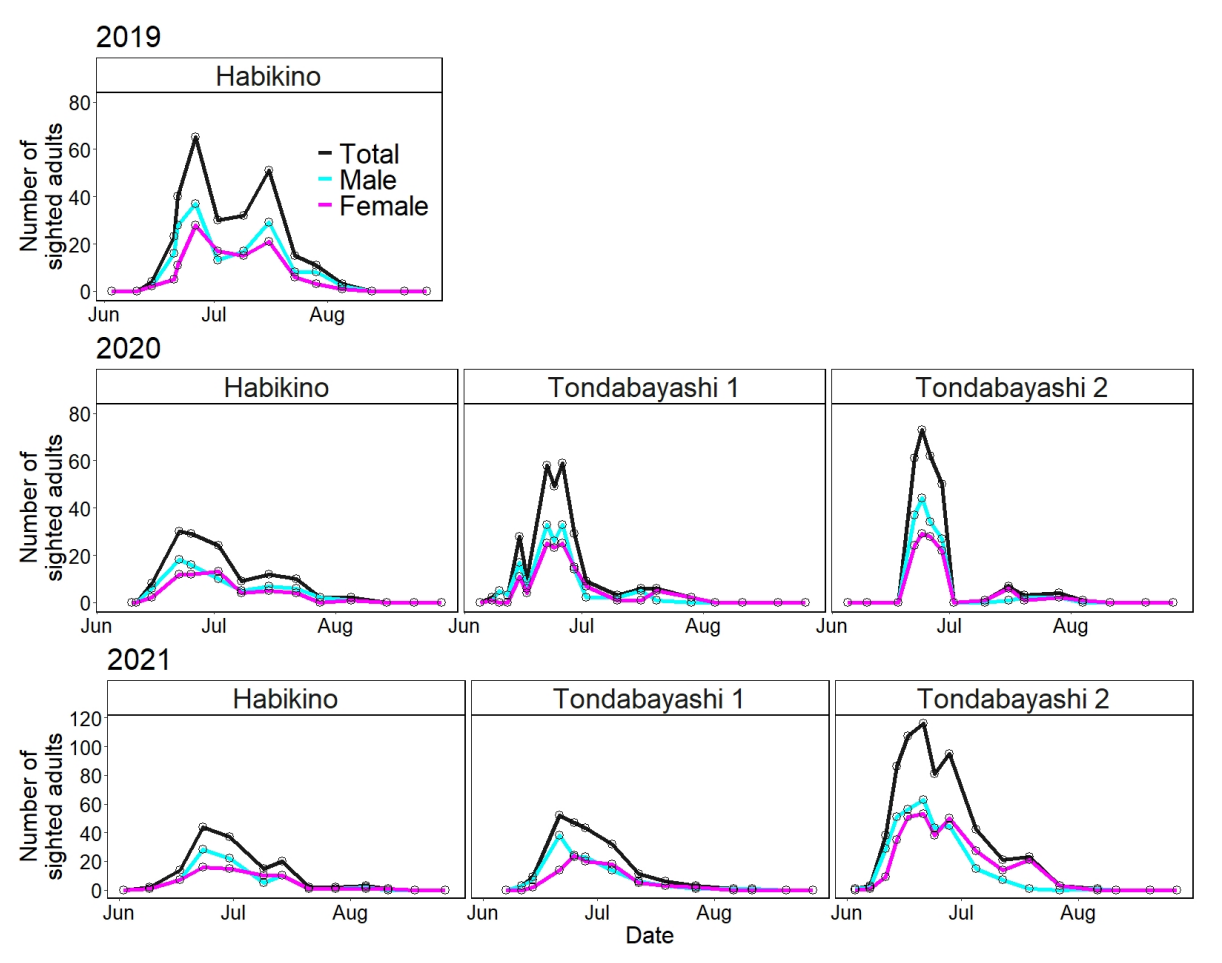
Line charts of the number of adults sighted in the field (total, male, and female) for each survey year and study site. Open circles represent the number of sighted adults on each survey date.

Figure 3 illustrates the number of *A. bungii* adults sighted on the monitoring trees per survey day for each survey year and study site using line charts from June to August. Table 1 shows the initial and last dates of sighting as well as the dates on which the highest number of adults were sighted.

Without considering the differences among the study sites, the duration of seasonal prevalence of *A. bungii* adults was from 14 June to 5 August in 2019, from 8 June to 5 August in 2020, and from 3 June to 11 August in 2021 (Table 1). The surveys conducted in 2021 showed that *A. bungii* adults could be detected for a duration of more than two months (June to August) in the field in Osaka Prefecture.

Regarding the initial sighting day of *A. bungii* adults at the survey site, the earliest and latest dates were 3 June (at Tondabayashi 2 in 2021) and 22 June (at Tondabayashi 2 in 2020), respectively (Table 1). The previous week’s survey on 22 June was not a suitable survey day (18 June; see Table S1 for climate data from the Japan Meteorological Agency, Sakai City: the nearest monitoring point to the study sites), and we did not identify an accurate initial sighting day in the 2020 survey. For the last sighting day, the earliest and latest dates were 29 July (at Tondabayashi 1 in 2020) and 11 August (at Tondabayashi 1 in 2021), respectively (Table 1). Regarding the peak dates, the earliest and latest dates were 21 June (at Tondabayashi 1 and 2 in 2021) and 26 June (at Habikino 1 in 2019 and at Tondabayashi 2 in 2020), respectively (Table 1). The peak dates varied slightly; however, the initial and last sighting dates were largely variable.

The period from the initial sighting day to the peak day was shorter than the period from the peak day to the last sighting day (e.g., in the 2019 survey; 12 days vs. 40 days); therefore, the distribution based on the number of sighted adults per day was not normal but skewed to the left (abundant in the early phase of seasonal prevalence) (Figure 3). In addition, there were very few or no adults in August for 3 years.

Regarding the sex ratio, it was male-biased from the initial sighting day to the peak day (Figure 3). The total number of sighted males was consistently higher than that of females in the field surveys (Table 1).

### 3.2. Adult Emergence from Infested Trees

#### 3.2.1. Dates of Adult Emergence from Infested Trees

**Figure 4:**
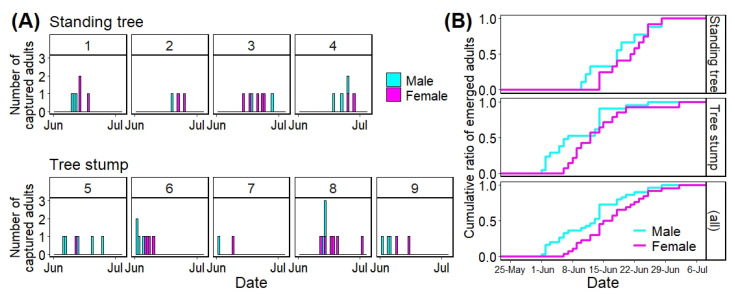
Number of adults that emerged daily from each tree (**A**) and cumulative ratio of the number of emerged adults among the tree types (including all trees) per day (**B**). Numbers (1 to 9) on each graph represent tree numbers that were allocated arbitrarily.

Figure 4 shows the number of *A. bungii* adults that emerged daily from each infested tree using stacked bar plots (A) and the cumulative ratio of the number of *A. bungii* adults that emerged from the infested trees via tree types and all trees using stepped line plots (B). Table 2 shows the initial and last emergence dates as well as the emergence period for each tree.

For the initial emergence dates of *A. bungii* adults from infested trees, the earliest and latest dates among the standing trees were 10 June (No. 1) and 19 June (No. 2), respectively, and those among the tree stumps were 1 June (No. 6) and 12 June (No. 8), respectively (Table 2). The maximum difference in the initial emergence dates was 18 days among all trees (No. 6 vs. No. 2). Regarding the last captured dates, the earliest and latest dates among the standing trees were 18 June (No. 1) and 28 June (No. 3 and 4), respectively, and those among the tree stumps were 9 June (No. 7) and 2 July (No. 8), respectively (Table 2). The total emergence duration for the trees was 31 days (No. 6 vs. No. 8).

The time to 50% adult emergence was 19 June in standing trees, 12 June in tree stumps, and June 14 in all trees (Figure 4B). These three dates were earlier than the peak date (23 June) in the field survey at Habikino in 2021 (Figure 4 and Table 1); by the peak day, 66.7% adults in standing trees, 94.3% adults in tree stumps, and 83.9% adults in all trees had emerged from the infested trees (Figure 4).

The emergence periods among the standing trees were between 7 (No. 2) and 15 days (No. 3), and those among tree stumps were between 8 (No. 7) and 21 days (No. 8) (Table 2).

Twenty-one adults (9 males and 12 females) emerged from 28 emergence holes (75.0%) in four standing trees; a total of 35 adults (21 males and 14 females) emerged from 52 holes (67.3%) in five tree stumps. There was no significant difference in the sex ratio between the tree types (standing tree vs. tree stump: Chi-squared test: *p* = 0.21) and no obvious trends based on tree type (Table 2).

#### 3.2.2. Factors Affecting Adult Emergence Day

Table 3 shows the results of the model selection on the LMM for adult-emergence days, including the regression coefficients of the selected variables in the models. The random intercept values for each test tree are shown in Appendix B (Table A2). The mean ± standard deviation (SD), min, max, and median of the forewing length of the tested insects are shown in Appendix C (Table A3).

The best model (LMM) for adult emergence days was the one that included the explanatory variables sex (coefficient = −3.684) and tree type (coefficient = −9.169) (Table 3). On comparing the marginal means of adult-emergence days between the sexes, we found a significant difference between males and females (Tukey HSD test, difference in the means = 3.684: female minus male, *p* = 0.003). This result suggests that males will emerge 3.684 days earlier than females. There was a significant difference between standing trees and tree stumps (Tukey HSD test, difference in the means = 9.169: standing tree minus stump, *p* = 0.003). This result suggests that adults in tree stumps will emerge 9.169 days earlier than those in standing trees.

The deviances of the selected LMM and that of the LM (each with the same explanatory variables) were 338.7 and 361.9, respectively. This indicates that the model with random intercept by trees can better explain the emergence days than the model considering no definition of statistical errors derived from trees.

## 4. Discussion

A timely pest-control strategy to reduce the abundance of *A. bungii* adults in the field can help mitigate damage to host trees. Our field surveys showed that the appearance period of *A. bungii* adults in Osaka Prefecture spanned >2 months from June to August, with peak sightings in late June (Table 1). Adults were abundant in the early phase of the appearance period and rare or absent in the late phase, as reflected in the distribution shapes of seasonal prevalence that skewed to the left with a unimodal peak in the field surveys (Figure 3). The same trend for adult field abundance during an appearance period was also reported in Tokushima Prefecture [9]. Therefore, control measures to suppress the population density of *A. bungii* adults in the field, such as insecticidal air-spraying, should be prioritized in the early phase of an appearance period; an appropriate application time in Osaka Prefecture is during the peak sighting in late June. To establish a control plan using insecticidal air-spraying, the application frequencies based on treatment concentrations need to be determined. Despite the increasing number of insecticides against *A. bungii* adults available in Japan [31], their residual effectiveness via treatment concentrations is not well examined. Studies should test the residual effects of insecticides and position each insecticidal treatment optimally in a pest control plan.

Among all the surveys, the seasonal prevalence of the Habikino 2019 survey appeared to have a bimodal peak—one in late June and the other in mid-July (Figure 3). Considering a general (2-year) life cycle in Japan [4], this bimodal peak may not necessarily suggest bivoltinism. The number of emerging *A. bungii* adults was higher on sunny days following a rainy day than on rainy days [32]. The second peak could correspond to a sunny day (16 July; see Table S1 for climate data from the Japan Meteorological Agency, Sakai City) and thus might be strongly influenced by the weather conditions.

The dates of initial appearance were earlier in Osaka Prefecture (except in the 2021 Tondabayashi 2 survey, which was unsuitable for identifying the initial detection date) than those previously recorded in other regions of Japan. The order of initial appearance in prefectures from earliest to latest dates is Osaka, Tokushima [8], Saitama [9], and Gunma [4]. In China, the appearance periods of *A. bungii* adults were earlier at lower latitudes [4]. From this perspective, we expected the appearance timing in Osaka Prefecture to be later than that in Tokushima Prefecture but obtained the opposite results. The annual climatic variations in each region [14] should also be considered. In the future, a meta-analysis using data from multiple years and various regions may help to clarify the factors influencing the seasonal prevalence of *A. bungii* adults.

The initial detection dates of *A. bungii* adults also differed among the surveys. In the 2021 surveys, the initial detection date of emerged adults from the infested trees (1 June) was earlier than that of sighted adults in the field (9 June) (Table 1 and Table 2); no adults were sighted on 2 June in the field survey. Sighting adults via personal observation in the field during the early phase of seasonal prevalence with low adult abundance was difficult. Therefore, a survey to capture emerging adults from infested trees may be more suitable than field surveys to determine the initial detection date. For the Japanese pine sawyer *Monochamus alternatus* Hope (Coleoptera: Cerambycidae), emergence-forecasting using infested logs has been attempted by calculating the effective accumulated temperature, based on both the larval developmental zero [10] and local average temperatures [33,34,35]. A larval-developmental zero has not yet been reported for *A. bungii*, and can be a topic for future research. An in-depth insight into *A. bungii* larvae may help accurately forecast the initial emergence dates based on average ambient temperatures in the field. Conversely, when identifying the appearance periods of *A.bungii* adults in the field, using infested trees may not be appropriate. This is because the appearance period in the survey of infested trees (from 1 June to 2 July) was shorter than that in the field survey (from 9 June to 11 August) (Table 1 and Table 2), leading to an underestimation of the period in the field.

As the best LMM suggested, tree types had a considerable influence on the adult emergence day, whereby adults emerged approximately 9.6 days earlier from tree stumps than from standing trees (Table 3). Additionally, the smaller deviance in the best LMM (338.7), with random error terms derived from trees, than that in the LM (361.9) suggests the influence of tree conditions. The maximum difference in the random intercept values was approximately 10.9 days (No. 1 vs. No. 8) among the trees (Appendix B). Thus, a very small number of samplings of infested trees or logs for emergence forecasting may hinder the identification of the initial emergence date. The factors affecting the difference between the tree types are unclear. In *Anoplophora glabripennis* (Motschulsky) (Coleoptera: Cerambycidae), higher temperatures facilitate faster larval and pupal development [36]. In this study, the tree stumps might have been exposed to more sunlight through gaps in the canopy created by tree-cutting [37], and thus, became heated more easily than standing trees, thereby accelerating larval development and adult emergence. The emergence speed of *A. bungii* could differ depending on the outdoor-net chamber where the infested logs were placed [26]; adult emergence was earlier in the net chamber without a roof than in that with a roof [26]. Therefore, a detailed survey is warranted to determine the factors influencing the emergence timings of *A. bungii* adults, such as temperature, host trees’ nutritional quality for larvae [38], and humidity in pupal chambers [39].

Regarding the emergence day by sex, males emerged approximately 3.7 days before females (Figure 4B) according to the most predictive model (LMM) (Table 3). This earlier emergence of *A. bungii* male is consistent with the finding of a previous study using infested logs [25] and with the overall ecological feature of the family Cerambycidae [4]. Moreover, our field surveys, based on the number of sighted adults, showed that the dates of initial and peak sighting in males were earlier than or equal to those in females (Figure 3).

The male/female ratio of *A. bungii* adults sighted in the field was male-biased (male/female >1) in total numbers, regardless of the survey years and study sites (Table 1). Therefore, *A. bungii* adults may be male-biased. However, our result is not consistent with that of previous studies. A survey using infested logs in Italy reported that the sex ratio was almost 1 (154/156) in *A. bungii* adults [3], similar to our result of emerged adults from infested trees (30/26). Another field survey of peach trees in Japan showed the same ratio: = of almost 1 (88/91) [8]. A survey using infested trees or logs appears to be the most reasonable method to determine the sex ratio because all emerged adults can be collected. Regarding the difference between the previous field survey [8] and our survey, the tree heights of the host species might affect the results; peach trees are usually <3 m and ornamental cherry trees are usually >3 m, the latter being our most monitored trees. Adult *A. bungii* males that emit a volatile sex-aggregating pheromone [19] may remain in the lower regions of trees for longer time than females, making it relatively easy to sight males and difficult to sight females on ornamental cherry trees. Future studies focusing on sex-specific locations on trees can help clarify the reason for the difference between our result and those of other studies.

In summary, based on a field survey conducted to determine the number of sighted *A. bungii* adults, the adult appearance period spanned >2 months (from June to August), with a peak sighting in late June in Osaka Prefecture. The distribution shape of the seasonal prevalence of *A. bungii* in the field was left-skewed, i.e., abundant in the initial phase and nearly absent by August. This abundance was indicated by a short-span emergence period of adults from the infested trees. A prediction model for the emergence days of *A. bungii* adults suggested that males emerge approximately 3.7 days earlier than females and that emergence days are largely influenced by infested-tree types (standing trees or tree stumps) and tree conditions. The sex ratio in the field survey using ornamental cherry trees was apparently male-biased; however, this result was inconsistent with previous studies, and differences between survey methods may have affected the results.

## Figures and Tables

**Table 1:** Survey dates and number of sighted adults by sex on the initial and last sighting days and highest number of sightings.

Survey Year	Study Site	Survey Date and Number of Sighted Adults by Sex (Male vs. Female)						Total Number of Sighted Adults
		Initial Sighting		Highest Number Sighting		Last Sighting		
2019	Habikino	14 Jun	4 (2|2)	26 Jun	65 (37|28)	5 Aug	3 (2|1)	274 (160|109|5)
2020	Habikino	15 Jun	8 (6|2)	22 Jun	30 (18|12)	5 Aug	2 (1|1)	126 (71|53|2)
	Tondabayashi 1	8 Jun	2 (1|1)	26 Jun	59 (33|25|1)	29 Jul	2 (0|2)	269 (148|120|1)
	Tondabayashi 2	22 Jun	61 (37|24)	24 Jun	73 (44|29)	4 Aug	1 (0|1)	262 (147|114|1)
2021	Habikino	9 Jun	2 (1|1)	23 Jun	44 (28|16)	11 Aug	1 (0|1)	140 (77|63)
	Tondabayashi 1	11 Jun	3 (3|0)	21 Jun	52 (38|14)	11 Aug	1 (1|0)	208 (120|88)
	Tondabayashi 2	3 Jun	1 (1|0)	21 Jun	116 (63|53)	6 Aug	1 (1|0)	617 (314|302|1)

Numbers in parentheses with vertical slashes represent the number of sighted males, females, and unknown sex (if applicable) in order. Adults with unknown sex had escaped before we could determine their sex.

**Table 2:** Survey on the initial and last capture dates, and the emergence period from a test tree.

Tree Type	Tree No.	First Capture		Last Capture		Emergence Period (Day)	Total Number of Adults	
		Date	Elapsed Day	Date	Elapsed Day		Emerged	Not Emerged
Standing tree	1	10 Jun	Day 10	18 Jun	Day 18	9	6 (3|3)	3
	2	19 Jun	Day 19	25 Jun	Day 25	7	3 (1|2)	1
	3	14 Jun	Day 14	28 Jun	Day 28	15	7 (2|5)	2
	4	18 Jun	Day 18	28 Jun	Day 28	11	5 (3|2)	1
Tree stump	5	6 Jun	Day 6	25 Jun	Day 25	20	10 (9|1)	8
	6	1 Jun	Day 1	10 Jun	Day 10	10	9 (5|4)	5
	7	2 Jun	Day 2	9 Jun	Day 9	8	2 (1|1)	1
	8	12 Jun	Day 12	2 Jul	Day 32	21	9 (3|6)	2
	9	2 Jun	Day 2	15 Jun	Day 15	14	5 (3|2)	1

Numbers in parenthesis with vertical slashes represent the number of emergence holes of male emergence and female emergence in order. The elapsed day was calculated based on the first emergence date of *Aromia bungii* adults (1 June in Tree No 6).

**Table 3:** Results of model selection (LMM) for adult emergence days.

Rank	AIC	Conditional R^2^	Regression Coefficient			
			Forewing Length	Sex (Female = 0)	Tree Type (Standing Tree = 0)	Intercept
1	348.7	0.706	–	−3.684	−9.169	21.365
2	350.5	0.709	0.154	−3.542	−9.145	17.670

Explanatory variables in the LMM formula (full model): forewing length + sex + tree type. AIC, Akaike’s information criterion; LMM, linear mixed model. “−” indicates that the variable is not chosen in the model.

## Data Availability

All data analyzed in this study are included in this article and supplementary materials.

## Supplementary Materials

The following supporting information can be downloaded at: https://www.mdpi.com/article/10.3390/insects13030222/s1, Table S1: climate data at Sakai City in Osaka Prefecture and number of adults sighted in the field surveys.

## Appendix A

**Table A1:** Number of monitoring trees at each study site.

Study Site	Tree Species	2019			2020			2021		
		Start	During Survey		Start	During Survey		Start	During Survey	
			Cut	No Access		Cut	No Access		Cut	No Access
Habikino	Ornamental cherry (*Cerasus* spp.)	138	0	0	87	0	0	91	0	0
	*Prunus mume*	27	0	0	27	0	0	44	0	0
	*Prunus cerasifera* var. atropurpurea	16	0	0	10	0	0	10	0	0
Tondabayashi 1	Ornamental cherry (*Cerasus* spp.)	―	―	―	244	10	0	231	11	0
Tondabayashi 2	Ornamental cherry (*Cerasus* spp.)	―	―	―	134	5	36	249	0	0

## Appendix B

**Table A2:** Result of random intercept values for each infested tree in the best linear mixed model (LMM) for adult emergence days in the 2021 survey.

Tree Type	Tree No.	Random Intercept Value
Standing tree	1	−5.424
	2	1.387
	3	0.351
	4	3.686
Tree stump	5	4.552
	6	−4.719
	7	−3.202
	8	5.513
	9	−2.143

Explanatory variables in the best LMM formula: forewing length + sex + tree type + (1 | Tree No.).

## Appendix C

**Table A3:** Mean ± standard deviation (SD), min, max, and median of forewing length of emerged adults from infested trees.

Sex	Tree Type	Mean ± SD	Min	Max	Median
Male	Standing tree	22.9 ± 2.2	18.7	25.1	23.7
	Tree stump	22.4 ± 1.9	18.8	26.3	22.2
Female	Standing tree	24.0 ± 1.5	20.8	26.5	23.9
	Tree stump	23.8 ± 1.7	20.0	25.7	24.3
